# An Expert-Supervised Registration Method for Multiparameter Description of the Knee Joint Using Serial Imaging

**DOI:** 10.3390/jcm11030548

**Published:** 2022-01-22

**Authors:** Hugo Babel, Patrick Omoumi, Killian Cosendey, Julien Stanovici, Hugues Cadas, Brigitte M. Jolles, Julien Favre

**Affiliations:** 1 Swiss BioMotion Lab, Lausanne University Hospital and University of Lausanne (CHUV-UNIL), CH-1011 Lausanne, Switzerland; hugo.babel@alumni.epfl.ch (H.B.); killian.cosendey@chuv.ch (K.C.); brigitte.jolles-haeberli@chuv.ch (B.M.J.); 2 Service of Diagnostic and Interventional Radiology, Lausanne University Hospital and University of Lausanne (CHUV-UNIL), CH-1011 Lausanne, Switzerland; patrick.omoumi@chuv.ch; 3 Department of Radiology, Cliniques Universitaires St Luc-UC Louvain, BE-1200 Brussels, Belgium; 4 Service of Orthopedics and Traumatology, Lausanne University Hospital and University of Lausanne (CHUV-UNIL), CH-1011 Lausanne, Switzerland; julien.stanovici@gmail.com; 5 Unité Facultaire d’Anatomie et de Morphologie, University of Lausanne (UNIL), CH-1005 Lausanne, Switzerland; hugues.cadas@unil.ch; 6 Institute of Microengineering, Ecole Polytechnique Fédérale Lausanne (EPFL), CH-1015 Lausanne, Switzerland

**Keywords:** computed tomography, magnetic resonance imaging, osteoarthritis, registration, relationships, segmentation, serial imaging

## Abstract

As knee osteoarthritis is a disease of the entire joint, our pathophysiological understanding could be improved by the characterization of the relationships among the knee components. Diverse quantitative parameters can be characterized using magnetic resonance imaging (MRI) and computed tomography (CT). However, a lack of methods for the coordinated measurement of multiple parameters hinders global analyses. This study aimed to design an expert-supervised registration method to facilitate multiparameter description using complementary image sets obtained by serial imaging. The method is based on three-dimensional tissue models positioned in the image sets of interest using manually placed attraction points. Two datasets, with 10 knees CT-scanned twice and 10 knees imaged by CT and MRI were used to assess the method when registering the distal femur and proximal tibia. The median interoperator registration errors, quantified using the mean absolute distance and Dice index, were ≤0.45 mm and ≥0.96 unit, respectively. These values differed by less than 0.1 mm and 0.005 units compared to the errors obtained with gold standard methods. In conclusion, an expert-supervised registration method was introduced. Its capacity to register the distal femur and proximal tibia supports further developments for multiparameter description of healthy and osteoarthritic knee joints, among other applications.

## 1. Introduction

Knee osteoarthritis is a painful, incapacitating joint disease affecting the quality of life of hundreds of millions of people worldwide [1,2,3]. Reducing this major socio-economic burden will notably require improving our understanding of the pathophysiology of the disease, as a prerequisite for earlier disease detection and more effective treatments. Recently, researchers have proposed enhancing the disease models by taking into account the relationships between tissue parameters, rather than studying the parameters in isolation [4,5]. To this end, there is a need for new developments to facilitate the multiparameter description of knee tissues.

Advances in medical imaging in the last decades have resulted in a vast panel of three-dimensional quantitative parameters that can be measured using magnetic resonance imaging (MRI) and computed tomography (CT) [6,7]. For example, particular MRI protocols can quantify cartilage morphology [8] or composition [9], and bone mineral density can be measured using CT [10]. Furthermore, it is possible to image the joint successively using different protocols and/or modalities, a procedure known as serial imaging, in order to extend the description of the tissues. Surprisingly, serial imaging has seldom been performed to characterize the relationships among parameters in knee osteoarthritis [11,12,13]. Additionally, to the authors’ knowledge, none of the studies on the relationships have registered images acquired with different protocols and/or modalities. In fact, the parameters were extracted independently from each set of images using regions of interest determined separately. While achieving multiparametric descriptions, this methodology suffers from some limitations. Specifically, consistency among the regions of interest is not guaranteed, the benefit of having complementary acquisition protocols allowing the identification of different tissues is underused, and processing time might be squandered by the replication of some operations with each image set [14,15,16,17,18]. Furthermore, although this methodology works for analyses based on large regions of interest [11,19,20,21], it prevents applications requiring higher resolution, for example, those analyzing the spatial variation of the parameters [10,22,23,24]. Consequently, relationship analyses would benefit from a registration among image sets.

A few authors have already registered knee images acquired with different protocols or modalities, mainly to help identify regions of interest that could not be (accurately) identified in some sets of images [25,26,27,28]. In these cases, the images were registered globally based on mutual information, such as voxel intensities [29,30]. Although common in medical imaging, this fully automatic approach is not always possible. Indeed, some acquisition protocols have been designed to capture the local properties of the knee and it would be risky to base the registration on the entire images without considering the specificities of the acquisition protocols. Therefore, there is a need for an alternative, expert-based, approach where the registration would be guided by an operator trained to identify specific features in the images allowing the registration to be completed. Advanced knee osteoarthritis analyses usually involve image segmentations in order to build three-dimensional mesh models of the tissues of interest. Thus, the registration could be done by positioning the three-dimensional models in the various image sets using features associated with the contour of the tissues. Recently, a method in this sense was proposed where femoral bone and cartilage models were built using an image set and then visually positioned by an operator in another image set where the tissue contours were only partially identifiable [31]. The method was shown to be reliable, supporting an expert-based approach for registration in serial imaging. However, this method, where the operator must manually update the translation and rotation values until the models are positioned correctly, could be improved by limiting the action of the operator to the placement of “attraction points” and having an algorithm automatically positioning the models according to the attraction points.

This study aimed to develop an expert-supervised registration method for serial imaging based on three-dimensional tissue models and attraction points. The method was evaluated for the registration of the distal femur and the proximal tibia, because there is a concrete need to combine image sets of the knee from complementary protocols or modalities to improve our understanding of knee osteoarthritis [4,5,6,7]. Specifically, the influence of the number of attraction points, as well as the intraprotocol and intermodality registration error were determined.

## 2. Materials and Methods

### 2.1. Expert-Supervised Registration Method

The method proposed in this study requires at least one set of images for the segmentation of the tissue of interest and the creation of its three-dimensional mesh model, and one or more other sets of images where the three-dimensional model is imported based on attraction points placed manually. The method works similarly with entire or partial tissues. In addition to single tissues, it is possible to work with an agglomeration of full and/or partial tissues. In the present study, there were two distinct (partial) tissues of interest: the distal femur and the proximal tibia. These anatomical parts correspond to the portions of the femur and tibia usually imaged in clinics for the evaluation of the knee joint [32]. To account for possible motion of the femur with respect to the tibia between image sets, the femur and tibia were registered separately.

**Figure 1:**
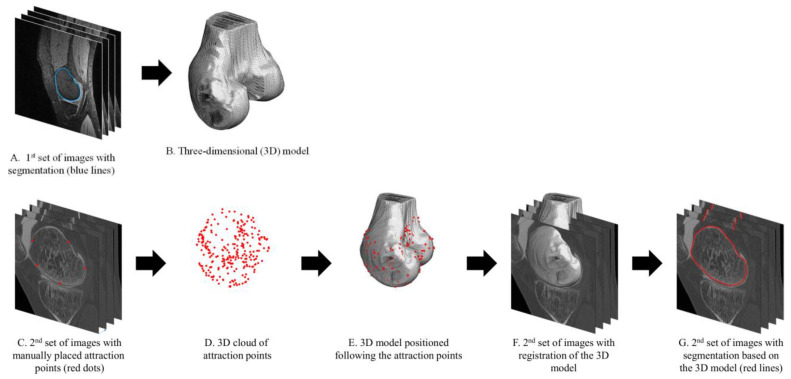
Illustration of the expert-supervised registration method for the distal femur. The method can accommodate other anatomical structures, such as the proximal tibia, and a higher number of image sets.

The procedure, which is similar for both bones, is illustrated for the femur in Figure 1. Specifically, previously described methods were used to segment and build a three-dimensional mesh model of the bones (Figure 1A,B) [32,33,34]. The segmentation followed a subpixel approach consisting of contouring the tissues in the sagittal plane with B-spline curves. A graphical interface was designed to load a set of images and let an operator navigate through the images in order to manually place attraction points on the edges of the bones (Figure 1C,D). To facilitate future use, the method was implemented to work with attraction points distributed more or less homogeneously in space. Concretely, with this methodology, the operator is simply asked to distribute the points, without having to localize particular features or follow more specific guidelines. After placement of the points for a bone model in an image set, the three-dimensional model was automatically positioned following its attraction points using the coherent point drift (CPD) algorithm (Figure 1E) [35]. At this stage, the procedure is completed, with the bone model from one image set registered to another image set (Figure 1F). The registration can be used to identify the bone voxels in the second image set (Figure 1G) or to establish an anatomical correspondence for more advanced analyses [27].

### 2.2. Imaging Datasets

Two datasets were used for this research project approved by the relevant ethics committees.

The first dataset corresponded to 10 formalin-fixed cadaveric adult knees scanned (DATA1_CT1) and rescanned (DATA1_CT2) after repositioning using a 64-row detector helical CT machine (Discovery CT750HD; GE Healthcare, Chicago, IL, USA). To allow the comparison of the proposed expert-supervised registration method to a gold standard, five fiducial markers were embedded in each distal femur and proximal tibia before scanning. The acquisition protocol was as follows: tube voltage, 120 kVp; reference tube current-time product, 200 mAs; bone convolution kernel, boneplus; voxel size of 0.5 mm × 0.5 mm × 0.312 mm. Following local regulations regarding research on deceased persons, the CT images were the only data available for the samples. A senior musculoskeletal radiologist with 11 years of experience (P.O.) read the CT scans and, based on the presence and severity of osteophytes [36], concluded that five of the knees had osteoarthritic changes (three mild and two severe changes). In addition, the CT images indicated that the knees were exempt of any signs of traumatic bone lesion, previous knee surgery, tumor, chronic inflammatory joint disease and articular crystal deposition disease.

The second dataset was a convenience sample of 10 knees from 10 individuals 50 years old or older with CT (DATA2_CT), MRI (DATA2_MRI) and radiography examinations performed on the same day. The CT scans in this dataset were obtained using a 40-row detector CT scanner (Somatom Definition AS; Siemens Healthcare, Forchheim, Germany). The acquisition protocol was: tube voltage, 120 kVp; reference tube current–time product, 350 mAs with application of a dose modulation protocol (Care Dose 4D; Siemens Healthcare, Forchheim, Germany); bone convolution kernel, U70u; voxel size of 0.3 mm × 0.3 mm × 0.3 mm. The MR images were acquired on a 3-Tesla device (Magnetom Verio, Siemens Healthcare, Erlangen, Germany), using a double echo steady state (DESS) sequence (repetition time (TR), 14.25 ms; echo time (TE), 5.09 ms; matrix size of 320 × 320; voxel size of 0.5 mm × 0.5 mm × 0.5 mm). The knees were from five males and five females with a median age of 62 years old (1st–3rd quartiles: 59–67). A grading [37] performed by a senior musculoskeletal radiologist (P.O.), based on lateral and posteroanterior weight-bearing radiographies, indicated that five of the knees were osteoarthritic (three moderately (Kellgren–Lawrence grade of 3) and two severely (Kellgren–Lawrence grade of 4)). In addition, the imaging evaluation indicated that the knees were exempt of any signs of traumatic bone lesion, previous knee surgery, tumor, chronic inflammatory joint disease and articular crystal deposition disease.

The femur and tibia were segmented in each acquisition and three-dimensional bone models were reconstructed, as explained above (see Section 2.1). One operator (H.B.) processed the knees in DATA1_CT1 and DATA2_MRI, whereas a second operator (K.C.) handled the knees in DATA1_CT2 and DATA2_CT. This allowed interoperator assessment, which is more relevant than intraoperator characterization [34].

### 2.3. Number of Attraction Points

The attraction points are a key element of the proposed expert-supervised registration method. Consequently, their influence should be determined to ensure that the method is used properly. As explained above (see Section 2.1), the method was designed to work with attraction points distributed more or less evenly in space. Therefore, the influence of the attraction points reduces to the question of the number of points. To characterize this aspect, one operator (H.B.) placed 4000 points on the edges of both the femur and tibia for each image set in DATA1_CT2. Then, the three-dimensional models obtained from the segmentation of DATA1_CT1 by the same operator were positioned in the DATA1_CT2 acquisitions using the proposed method. For each bone, the positioning was computed 10 times, with 32, 64, 128, 256, 512 and 1024 randomly selected attraction points, respectively. The 600 (10 knees × 6 numbers of points × 10 repeats) femoral and tibial models obtained this way in the images sets of DATA1_CT2 were compared to the models from the segmentation of DATA1_CT2 by the second operator (K.C.), considered as references. Two common metrics were used to compare the registered and reference models: (1) the mean absolute distance (MAD), which indicates how much the surface of one model differs compared to the surface of the other model [38]; (2) the Dice index, which quantifies the overlap between the volumes included in each model [39]. Both metrics were computed in three dimensions, and expressed in millimeters for the MAD and as a ratio varying between 0.0 (no overlap) and 1.0 (perfect overlap) for the Dice index. Finally, Wilcoxon rank-sum tests [40], with a Bonferroni correction for multiple comparisons, were performed to compare the MAD and Dice index between consecutive numbers of points. This analysis allowed the definition of the ideal number of attraction points that was used in the rest of the study.

### 2.4. Intraprotocol Registration Error

While the proposed expert-supervised method will fully reveal its usefulness in interprotocol or intermodality registrations, an intraprotocol assessment is instructive because it provides a basis evaluation independently of the differences among the acquisition protocols or modalities. To assess the registration error intraprotocol, the three-dimensional bone models from the segmentation of DATA1_CT1 by one operator (H.B.) were imported in the DATA1_CT2 images sets and compared to the bone models from the segmentation of DATA1_CT2 by the second operator (K.C.) that served as reference models. To allow a pertinent interpretation of the results, three registration methods were tested. First, the bone models were positioned using the proposed expert-supervised method based on an ideal number of attraction points (see Section 2.3) placed by the operator who segmented the acquisitions in DATA1_CT1 (H.B.). Second, the bone models were positioned by overlapping the fiducial markers that were embedded in the bones before CT scanning [41]. This method represents the gold standard, but it is rarely possible in vivo and interprotocol or intermodality. Therefore, to allow the assessment of the method in an intermodality setting (see Section 2.5), a substitution gold standard was also considered, where the bone models were positioned by registration of the entire bone models using a standard algorithm [35]. This third method corresponds to an extreme version of the proposed method, where the operator would place an excessive number of attraction points. In practice, the third method is obviously suboptimal as it would require unnecessary segmentation work and would not be applicable to acquisition protocols allowing only partial segmentation of the tissues. For the three methods, the registered bone models of the 10 femurs and tibias in DATA1 were compared to the reference bone models using the MAD and Dice index. Lastly, to evaluate the proposed expert-supervised method and the substitution gold standard method, the MAD and Dice index values obtained with these methods were compared to the results of the gold standard method using Wilcoxon signed rank tests [40].

### 2.5. Intermodality Registration Error

The intermodality registration error was evaluated similarly to the intraprotocol error by having one operator (H.B.) segment one dataset (DATA2_MRI) and import the bone models obtained that way into another dataset (DATA2_CT). The other dataset was processed by a second operator (K.C.), leading to reference bone models that could be compared to the registered models using the MAD and Dice index. Differently from the intraprotocol assessment, this time, MRI-based models were positioned in CT images. Moreover, since using fiducial markers was impossible, only two registration methods were considered, and the MAD and Dice index values of the proposed expert-supervised method were compared to the values obtained with the substitution gold standard method using Wilcoxon signed rank tests [40].

All processing and statistical analyses were performed using Matlab R2019b (Mathworks, Natick, MA, USA). An alpha-level set a priori at 5% was used to determine statistical significance.

## 3. Results

### 3.1. Number of Attraction Points

**Figure 2:**
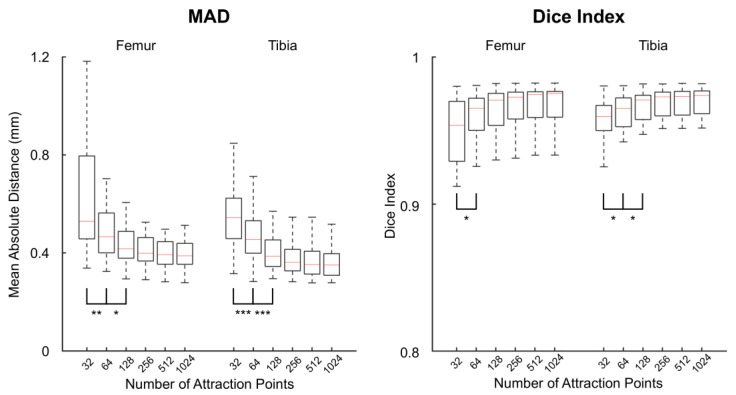
Boxplots of the mean absolute distances (MAD, **left**) and Dice indices (**right**) between the registered and reference bone models for varying numbers of attraction points. Asterisks indicate statistically significant differences between successive numbers of points (*: adjusted *p* < 0.05, **: adjusted *p* < 0.01, ***: adjusted *p* < 0.001).

The median MAD between the registered and reference models decreased from 0.53 mm with 32 points to 0.39 mm with 1024 points in the femur, and from 0.54 mm with 32 points to 0.35 mm with 1024 points in the tibia (Figure 2, Table 1). Differences in MAD achieved statistical significance between 32 and 64 points, as well as between 64 and 128 points, both in the femur and the tibia (adjusted *p* ≤ 0.03). For both bones, the median Dice indices were above 0.95 with the six numbers of points. Dice indices differed significantly between 32 and 64 points in the femur (adjusted *p* = 0.03), and between 32 and 64 points as well as between 64 and 128 points in the tibia (adjusted *p* ≤ 0.04).

Based on the statistically significant differences, the ideal number of attraction points was defined as 128.

### 3.2. Intraprotocol Registration Error

**Figure 3:**
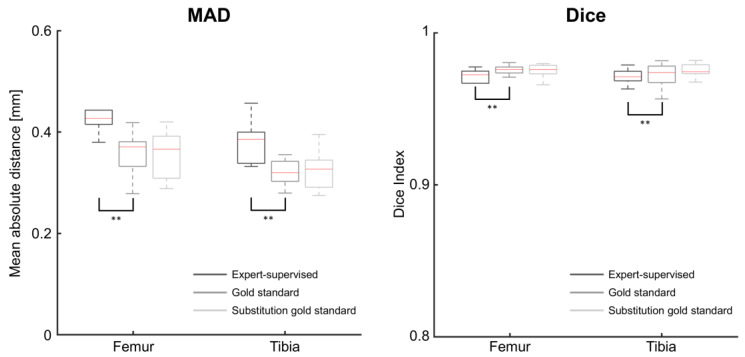
Boxplot of the mean absolute distances (MAD, **left**) and Dice indices (**right**) between the registered and reference bone models for three registration methods used intraprotocol. The expert-supervised method was run with 128 attraction points. Asterisks indicate statistically significant differences between methods (**: *p* < 0.01).

The intraprotocol results are presented in Figure 3 and Table 2. Before describing them in detail, it is useful to recall that this study assessed the methods using an interoperator setting. With this setting, the MAD and Dice indices are sensitive to the different segmentations produced by the two operators. These segmentation differences explain why the MAD and Dice indices obtained with the gold standard do not indicate perfect matching. This being said, the results in Figure 3 and Table 2 are twofold.

First, the proposed expert-supervised method resulted in a statistically significantly larger MAD (*p* = 0.002) and lower Dice indices (*p ≤* 0.004) than the gold standard. In the median, the MAD differed by 0.06 mm and the Dice indices by 0.003 unit.

Second, the MAD and Dice indices differed, in the median, by less than 0.01 mm and less than 0.001 unit between the gold standard and its substitution, respectively. There was no statistically significant difference between these two methods.

### 3.3. Intermodality Registration Error

**Figure 4:**
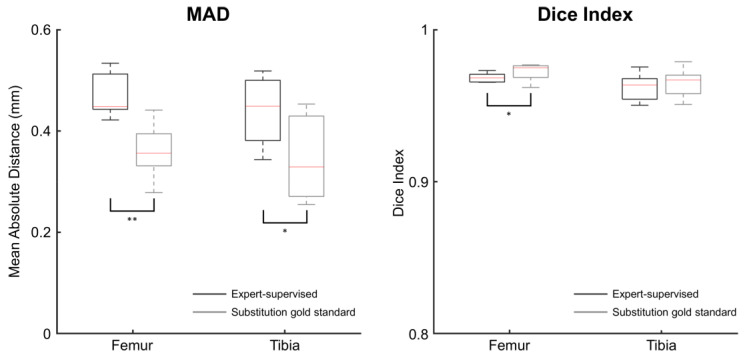
Boxplot of the mean absolute distances (MAD, **left**) and Dice indices (**right**) between the registered and reference bone models for two registration methods used intermodality. The expert-supervised method was run with 128 attraction points. Asterisks indicate statistically significant differences between methods (*: *p* < 0.05, **: *p* < 0.01).

When used to register MRI-based bone models in CT image sets, in an interoperator setting, the proposed expert-supervised method reported statistically significantly larger MAD than the substitution gold standard method, in the median, by 0.08 mm for the femur (*p* = 0.003) and 0.07 mm for the tibia (*p* = 0.014) (Figure 4, Table 3). Dice indices were statistically significantly lower with the proposed method for the femur (*p* = 0.045), whereas no statistical difference existed for the tibia (*p* = 0.427). In the median, the differences in Dice indices were of 0.004 unit for the femur and 0.003 unit for the tibia.

## 4. Discussion

This study presented an expert-supervised registration method and showed its capacity to register the distal femur and proximal tibia. This new method is particularly interesting because it relies on the expertise of an operator, without requiring the operator to do the entire job manually. The adaptability it confers, with the operator specifying the features (attraction points) to consider in the images, could be a significant advantage with acquisition protocols optimized to measure particular parameters and not trustable over the entire images [42]. While the method was primarily designed to bring novel possibilities for multiparameter descriptions of the knee joint in future works [4,5], it could certainly prove useful for a range of pathologies that would benefit from a more comprehensive characterization of the tissues using serial imaging, including oncological, inflammatory, and metabolic applications [43,44,45,46].

Establishing a spatial correspondence among image sets acquired with different protocols and/or modalities, as proposed in this study, offers several advantages. First, it could enhance the quality of the three-dimensional tissue models by limiting the segmentation to the image sets offering the most distinct contours. Working with image sets easier to segment could also prove beneficial for the use of automatic segmentation [47,48,49]. Second, placing attraction points in some image sets rather than segmenting all the image sets could save a significant amount of time. The gain could become even more appreciable when some image sets display poor contours. Third, registering the models could enhance the analysis thanks to a better spatial consistency among the image sets and the possibility to perform more advanced analyses, for example in terms of spatial variations [10,22,23,24].

As expected, the registration errors decreased with a larger number of attraction points. However, there was a plateau starting at about 128 points, confirming the practicability of the method. Indeed, less than three minutes were required to place 128 points (256 points were placed in less than five minutes). Statistically significant differences were observed between the proposed method and the gold standard/substitution gold standard, but they were too small to be clinically relevant, with differences below 0.1 mm in median MAD and 0.005 unit in median Dice indices. It is also worth noting that although the substitution gold standard is frequently used in literature [50], to the authors’ knowledge, it was never assessed for distal femur and proximal tibia registrations. In this regard, the present study confirmed its validity, at least with acquisition protocols providing clear bone contours. The registration of knee tissues being a relatively new consideration, there are only few prior data for comparison. Nevertheless, the median MAD (≤0.45 mm) and Dice indices (≥0.96) obtained with the proposed method both intraprotocol and intermodality were in line with previous studies reporting precision errors between 0.48 and 0.81 mm [51] and Dice indices between 0.96 and 0.98 [27,52] for the distal femur and proximal tibia. While this comparison should be interpreted with caution due to numerous experimental differences among studies, such as the number and condition of the study knees, the acquisition protocols and the intra/interoperator settings, it suggests that the proposed method based on attraction points could perform at least equivalently to other methods requiring full segmentation of the bones.

Basing the registration on attraction points is interesting because it does not require preprocessing the image sets to have a specific field of view or resolution. In addition to improving the accuracy of the data extracted from the images after registration and speeding up computation time [29], working directly with the original data makes the method compatible with a larger panel of acquisition protocols and modalities. This could prove particularly relevant for a democratization of analyses based on registration in clinics, as routine acquisition protocols can include anisotropic resolutions or partial imaging of the joint.

It should be noted that, beyond a specific method, the present study introduced a registration concept for serial imaging. In fact, the idea of positioning a three-dimensional model using attraction points could be implemented using other segmentation, model building and distance minimizing algorithms [29]. In order to assess the registration error, this study focused on the distal femur and proximal tibia. The method is obviously also applicable to other tissues and image sets where only a portion of the tissue contour is identifiable. Once the position of a tissue model is known in two or more image sets, a spatial correspondence exists among the image sets. This correspondence could be used to register other tissues, or more generally to register voxels [53,54]. For example, a bone and cartilage model could be created with one image set and then, by positioning the model based on the bone contour, it could be possible to identify the cartilage in another image set where the cartilage edges are hardly identifiable [31].

Following the study objectives, this work assessed the influence of the number of attraction points and the registration error. While this was done appropriately, with an adequate number of knees representative of the general population [55,56,57,58] and by having the knees processed by two operators with typical levels of experience, further research will be necessary to characterize the proposed method in context. The limited information available for the study knees did not affect the method or the results presented in this article. However, future applications of the method might require a more specific description of the samples.

## 5. Conclusions

In conclusion, this study presented an expert-supervised registration method and showed its capacity to register the distal femur and proximal tibia. By facilitating multi-parameter description, in the future, this novel method could contribute to a better understanding of healthy and osteoarthritic knee joints with possible implications for disease management, such as earlier detection. The expert-supervised registration concept could certainly prove useful for other applications that would benefit from a more comprehensive characterization of the tissues using serial imaging.

## Figures and Tables

**Table 1:** Mean absolute distances (MAD) and Dice indices between the registered and reference bone models for varying numbers of attraction points.

		Number of Attraction Points					
		32	64	128	256	512	1024
Femur	MAD	0.53 ^A^	0.47 ^A,b^	0.42 ^b^	0.4	0.39	0.39
		(0.46, 0.80)	(0.40, 0.56)	(0.38, 0.49)	(0.37, 0.46)	(0.35, 0.45)	(0.35, 0.44)
	Dice index	0.95 ^a^	0.97 ^a^	0.97	0.97	0.97	0.98
		(0.93, 0.97)	(0.95, 0.97)	(0.95, 0.98)	(0.96, 0.98)	(0.96, 0.98)	(0.96, 0.98)
Tibia	MAD	0.54 **^A^**	0.45 **^A,B^**	0.39 **^B^**	0.36	0.35	0.35
		(0.46, 0.62)	(0.40, 0.53)	(0.34, 0.45)	(0.33, 0.41)	(0.31, 0.41)	(0.31, 0.40)
	Dice index	0.96 ^a^	0.97 ^a,b^	0.97 ^b^	0.97	0.97	0.97
		(0.95, 0.97)	(0.95, 0.97)	(0.96, 0.97)	(0.96, 0.98)	(0.96, 0.98)	(0.96, 0.98)

MAD and Dice indices are presented as the median (1st quartile, 3rd quartile) of 100 registrations (10 knees × 10 repeats). MAD are reported in mm and Dice indices are unitless. Superscript letters indicate statistically significant differences between successive numbers of points (lowercase: adjusted *p* < 0.05, uppercase: adjusted *p* < 0.01, bold uppercase: adjusted *p* < 0.001). Letters ^a, A and **A**^ correspond to differences between 32 and 64 points, whereas letters ^b, B and **B**^ correspond to differences between 64 and 128 points.

**Table 2:** Mean absolute distances (MAD) and Dice indices between the registered and reference bone models for three registration methods used intraprotocol.

		Expert-Supervised	Gold Standard	Substitution Gold Standard
Femur	MAD	0.43 (0.41, 0.44) **	0.37 (0.33, 0.38)	0.37 (0.31, 0.39)
	Dice index	0.97 (0.97, 0.97) **	0.98 (0.97, 0.98)	0.98 (0.97, 0.98)
Tibia	MAD	0.39 (0.34, 0.40) **	0.32 (0.30, 0.34)	0.33 (0.29, 0.34)
	Dice index	0.97 (0.97, 0.98) **	0.97 (0.97, 0.98)	0.97 (0.97, 0.98)

Data are presented as median [1st quartile, 3rd quartile] over 10 knees. MAD are reported in mm and Dice indices are unitless. The expert-supervised method was run with 128 attraction points. Asterisks indicate statistically significant differences with respect to the gold standard (**: *p* < 0.01).

**Table 3:** Mean absolute distances (MAD) and Dice indices between the registered and reference bone models for two registration methods used intermodality.

		Expert-Supervised	Substitution Gold Standard
Femur	MAD **	0.45 (0.44, 0.51)	0.36 (0.33, 0.39)
	Dice index *	0.97 (0.97, 0.97)	0.97 (0.97, 0.98)
Tibia	MAD *	0.45 (0.38, 0.50)	0.33 (0.27, 0.43)
	Dice index	0.96 (0.95, 0.97)	0.97 (0.96, 0.97)

Data are presented as median [1st quartile, 3rd quartile] over 10 knees. MAD are reported in mm and Dice indices are unitless. The expert-supervised method was run with 128 attraction points. Asterisks indicate statistically significant differences between the two methods (*: *p* < 0.05, **: *p* < 0.01).

## Data Availability

The data are not publicly available due to regulatory provisions.
